# Comparison of highest and overall percentage Gleason pattern 4 in prostate cancer biopsies

**DOI:** 10.1007/s00428-025-04117-2

**Published:** 2025-06-06

**Authors:** L. J. Kroon, K. P. Leeuwenburgh, S. Remmers, C. F. Kweldam, R. C. N. van den Bergh, C. H. Bangma, G. J. L. H. van Leenders

**Affiliations:** 1 Department of Pathology, Erasmus MC Cancer Institute, University Medical Center Rotterdam, Rotterdam, The Netherlands; 2 Department of Urology, Erasmus MC Cancer Institute, University Medical Center, Na-15, P.O. Box 2040, 3000 CA Rotterdam, The Netherlands; 3 Anser Prostate Network, Rotterdam, The Netherlands

**Keywords:** Adverse pathology, Carcinoma, Cribriform, Gleason, Prostate biopsy, Prostate cancer, Radical prostatectomy

## Abstract

Current guidelines recommend pathologists to report percentage Gleason pattern 4 (GP4%) in Gleason score (GS) 7 prostate cancer (PCa) biopsies. However, it is unspecified whether the highest or overall GP4% should be reported. This study aims to clarify which quantification method correlates best with radical prostatectomy (RP) pathology. This study included 308 men with the highest GS 3 + 4 = 7, 4 + 3 = 7, or 4 + 4 = 8 on centrally revised systematic and/or targeted biopsies who underwent RP between 2018 and 2022. The highest and overall biopsy GP4% were compared with RP GP4% using Spearman’s rank correlation coefficient and adverse pathology (AP) (pT-stage ≥ T3, GS ≥ 4 + 3 = 7 and/or pN1) using multivariable logistic regression models adjusted for clinical tumor stage, prostate specific antigen (PSA), percentage of tumor positive biopsies, biopsy modality (systematic/targeted/both), and cribriform pattern. Both quantification methods correlated with RP GP4% (both rho = 0.59), and no significant difference was found between them (*p* = 0.78). On multivariable analyses, both GP4% quantification methods were significantly associated with AP (per 10% increase, highest GP4% odds ratio [OR] 1.26 [95% CI 1.14–1.39], overall GP4% OR 1.38 [95% CI 1.22–1.58], both *p* < 0.001). The area under the curve (AUC) was slightly better for overall (0.78 [95% CI 0.73–0.83]) than the highest GP4% (0.76 [95% CI 0.71–0.81], *p* = 0.041). This study found that the highest and overall biopsy GP4% both correlated with RP GP4%. Although the discriminative performance of the highest and overall GP4% was comparable with respect to AP at RP, the overall GP4% statistically slightly outperformed the highest GP4%. Including the overall GP4% may have added value in risk stratification and clinical decision-making in a subset of PCa patients.

## Introduction

The Gleason score (GS) is the pathological cornerstone for risk stratification in prostate cancer (PCa) patients. This grading system, which is based on the microscopic assessment of PCa growth patterns, provides prognostic information, guides treatment decisions, and predicts oncological outcomes. Currently, the International Society of Urological Pathology (ISUP), Genitourinary Pathology Society (GUPS), and European Association of Urology (EAU) recommend reporting the GS of all prostate biopsy sites separately and, optionally, an overall GS of all biopsy sites combined [1–3].

There is a need for further sub-classification of patients with GS7 PCa, who exhibit heterogeneous outcomes, to improve risk stratification and avoid over- or under-treatment [4, 5]. By distinguishing GS7 into 3 + 4 = 7 (Grade group [GG] 2) and 4 + 3 = 7 (GG3), the proportion of Gleason pattern 4 (GP4) is integrated into patient management algorithms. Several studies have suggested that further quantification of GP4 yields additional prognostic information [6–8]. Therefore, ISUP, GUPS, World Health Organization (WHO), College of American Pathologists, and the International Collaboration of Cancer Reporting (ICCR) all recommend reporting GP4 percentage (GP4%) for GS7 in prostate biopsies [2, 3, 9–11]. The exact method of GP4% reporting has, however, not been specified. Commonly used methods include reporting GP4% for each individual biopsy site, the highest GP4% of any biopsy site, or a global/overall GP4% over all biopsy sites. This variability may lead to inconsistency in reporting practices and clinical decision-making. Therefore, the aim of this study is to compare the prognostic value of the PCa biopsy highest and overall GP4% with findings at RP.

## Methods

### Patient selection

This study included patients who underwent a robot-assisted RP between September 2018 and August 2022 within the Anser Prostate Network. The Anser Prostate Network is a regional collaboration between eight medical centers in The Netherlands, which refer all patients scheduled for RP to one high-volume operation center, the Anser Prostate Operation Clinic located at the Maasstad Hospital, Rotterdam. The diagnostic work-up, including biopsy assessment, imaging, and multidisciplinary team meetings, is mostly performed in the referring centers. Data were prospectively collected and retrospectively analyzed. We included patients with the following criteria: provided informed consent; revised biopsy highest GS 3 + 4 = 7, 4 + 3 = 7, or 4 + 4 = 8; and availability of the RP pathological report. Men with targeted and/or systematic biopsies were included. We excluded men who only had positive cores on one site (systematic left/systematic right/targeted lesion) because, for those men, the highest and overall GS and GP4% are identical by definition. The study was approved by the institutional ethics board of Erasmus MC (METC-2019–0352) and the individual Anser Prostate Network centers.

### Pathological assessment

**Fig. 1:**
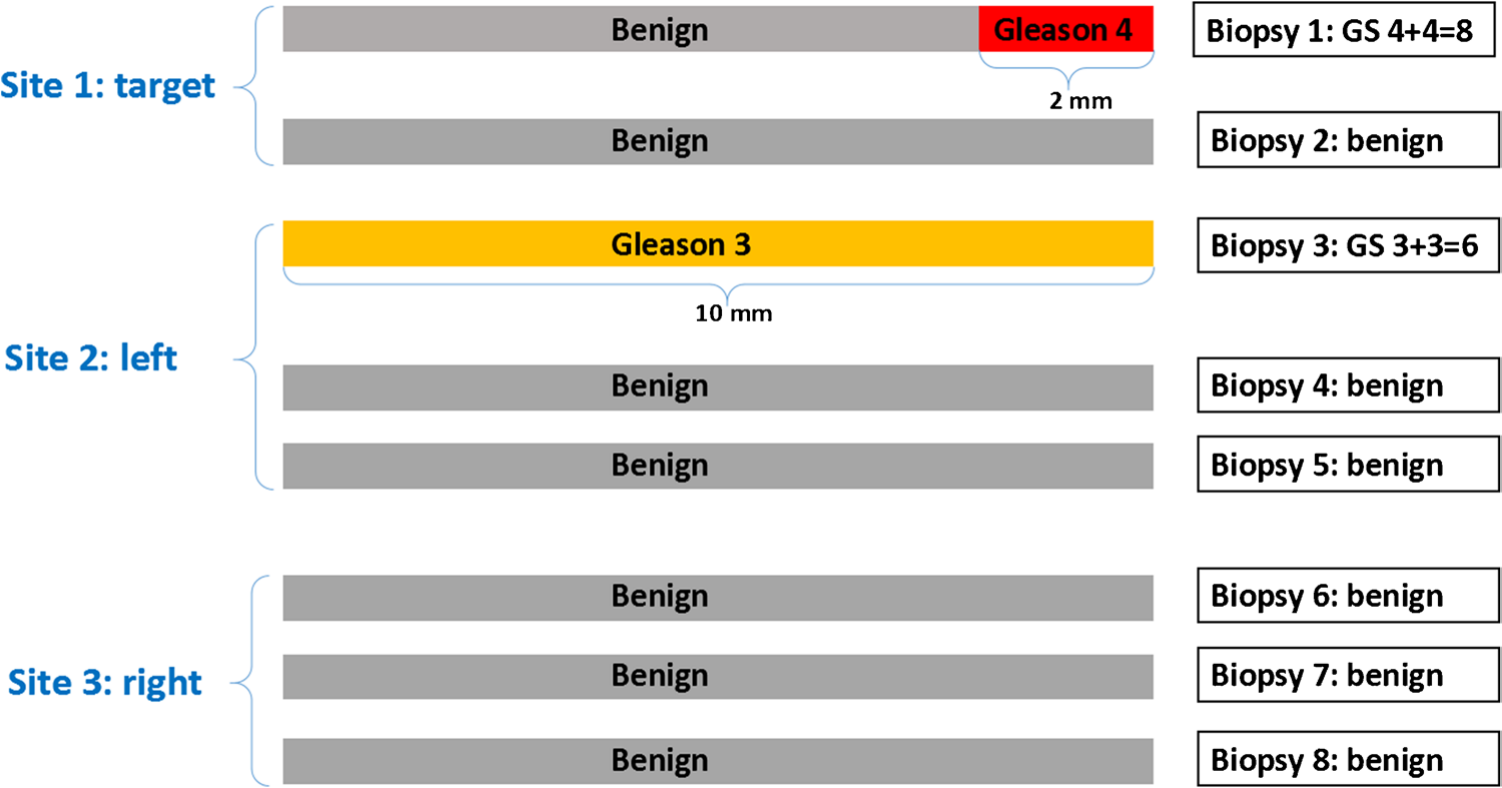
Example of prostate cancer biopsy assessment. This example includes 8 biopsy cores: 2 target cores (site 1), 3 systematic cores left (site 2) and 3 systematic cores right (site 3). In site 1, one core has 2 mm of Gleason pattern 4, diagnosed as GS 4 + 4 = 8 with 100% GP4. In site 2, one core has 10 mm Gleason pattern 3, diagnosed as GS 3 + 3 = 6 with 0% GP4. In site 3, all cores were benign. In this example, the highest GP4% is 100% and the overall GP4% is 17% (2 mm/12 mm). The highest GS is 4 + 4 = 8 and the overall GS is 3 + 4 = 7

All prostate biopsies were reviewed for study purposes by one genitourinary pathologist (GvL) and graded according to ISUP 2019 recommendations [2]. Biopsies were assessed by site in either left systematic, right systematic, or target lesions. Each site generally encompassed the mean of several biopsy cores. For each site, the GP4%, total cumulative length of carcinoma, and the presence of invasive cribriform (CR) and/or intraductal carcinoma (IDC) was recorded. No distinction was made between CR and IDC because they show overlapping morphological features and are difficult to distinguish without basal cell immunohistochemistry. The biopsy site with the highest GS was defined as the biopsy site containing the highest GP4%. Overall GP4% was determined by calculating the sum of absolute GP4 length over all cores in millimeters divided by total tumor length (Fig. 1). RPs were formalin-fixed, completely embedded, and assessed according to current ISUP grading recommendations by pathologists of the operating center who frequently held sessions to limit inter-observer variability [12]. RP specimens were not revised for study purposes.

### Study outcome

Study outcomes of interest were GP4% and adverse pathology (AP) at RP. AP was defined as at least one of the following pathological features at RP: pathological tumor stage (pT-stage) T3 or higher, GS 4 + 3 = 7 (GG3) or higher, and/or lymph node metastasis (pN1) at pelvic lymph node dissection (PLND) [13]. If multiple tumors were present at RP, GP4% of the index lesion was used.

### Statistical analysis

Variable distributions were visually inspected and expressed in median and interquartile range (IQR). We evaluated the difference between the overall and highest GS at biopsy, as well as the concordance of GG at RP. We used Spearman’s rank correlation coefficient (rho) to assess the correlation between GP4% on biopsy and RP. To test for statistically significant differences between rhos, Fisher’s z-transformation was used [14]. Uni- and multivariable logistic regressions were performed to assess the relationship between biopsy quantification methods and AP at RP. The multivariable model was adjusted for clinical tumor (cT) stage (levels: T1/T2/T3), pre-operative prostate specific antigen (PSA), percentage of tumor-positive biopsies, biopsy modality (levels: systematic only/target only/both systematic and target), and CR/IDC. CR/IDC was added to the model, as previous studies had shown CR/IDC is a prognostic predictor for AP [15, 16]. We used area under the curve (AUC) to compare the discriminative ability of both quantification methods. Since the models were not nested, we compared the AUCs with the DeLong test. A *p*-value of ≤ 0.05 was considered statistically significant. We used R version 4.3.2 for all statistical analyses.

## Results

### Patient characteristics

In total, 308 patients were included (Table 1). Their median age was 68 years (IQR 63–71) and median PSA was 9.7 ng/ml (IQR 6.4–13.4). Sixty-four percent of patients had palpable disease at rectal examination, and the median number of biopsy cores taken was 10 (IQR 8–12). In 63% of patients, the highest biopsy GS was 3 + 4 = 7; in 30%, it was 4 + 3 = 7; and in 7%, it was 4 + 4 = 8. The median overall GP4% was 24% (IQR 8.6–55), and the median GP4% of the highest site was 30% (IQR 10–70). At RP, 61% of patients had AP; 37% had GG ≥ 3, 48% had pT3, and positive nodal stage was found in 14%.

**Table 1:** Patient characteristics. Values are displayed as median (interquartile range) or frequency (percentage)

Patient characteristics	*N* = 308
Age (years)	68 (63, 71)
PSA (ng/ml)	9.7 (6.4, 13.4)
Clinical tumor stage	
T1	112 (36)
T2	128 (42)
T3	68 (22)
Systematic biopsies	287 (93)
Target biopsies	156 (51)
Target and systematic biopsies	135 (44)
Total biopsy cores	10 (8, 12)
Overall total length GP4 (mm)	5.6 (2.0, 17)
Overall total tumor length (mm)	30 (19, 47)
Overall GP4%	24 (8.6, 55)
Highest biopsy site total tumor length (mm)	17.3 (9.0, 27)
Highest biopsy site GP4 (mm) length	3.9 (1.6, 12)
Highest site GP4%	30 (10, 70)
CR/IDC presence at biopsy	157 (51)
CR/IDC presence at RP	203 (66)
GG at RP	
GG1	10 (3.2)
GG2	183 (59)
GG3	92 (30)
GG4	16 (5.2)
GG5	7 (2.3)
pT-stage at RP	
T2	160 (52)
T3a	95 (31)
T3b	53 (17)
Adverse pathology	188 (61)
PLND performed	218 (71)
pN1	42 (14)

*PSA* prostate specific antigen, *RP* radical prostatectomy, *GP4* Gleason pattern 4, *GP4%* Gleason pattern 4 percentage, *GG* grade group, *CR/IDC* cribriform growth or intraductal carcinoma, *PLND* pelvic lymph node dissection, *pN1* positive lymph node found at PLND

### Concordance of highest and overall GS at biopsy

The overall and highest GS were the same in 89% of patients (Table 2). Concordance between biopsy and RP GGs was 67% for the overall biopsy and 63% for the highest biopsy GG. Using the highest biopsy GS, more patients were downgraded at RP (20%) than using overall GS (13%). This effect was mainly due to patients who downgraded from 4 + 3 = 7 on the highest biopsy to 3 + 4 = 7 at RP. Upgrading was seen in 20% with respect to overall biopsy and 17% with the highest biopsy GS, mainly due to biopsy 3 + 4 = 7 being upgraded to 4 + 3 = 7 at RP.

**Table 2:** Concordance between Gleason score at biopsy and RP using highest and overall biopsy GP4%. Values are displayed as frequencies

*Overall biopsy *	*Highest biopsy Gleason score*					
*Gleason score*	**3 + 4 = 7**		**4 + 3 = 7**	**4 + 4 = 8**		*Total*
*3* + *4* = *7*	193		22	2		217
*4* + *3* = *7*	0		71	10		81
*4* + *4* = *8*	0		0	10		10
*Total*	193		93	22		308
***Overall biopsy Gleason score***	***RP GG***					
	**GG1**	**GG2**	**GG3**	**GG4**	**GG5**	Total
*3* + *4* = *7*	9	159	41	6	2	217
*4* + *3* = *7*	1	24	44	7	5	81
*4* + *4* = *8*	0	0	7	3	0	10
*Total*	10	183	92	16	7	308
***Highest biopsy Gleason score***	RP GG					
	**GG1**	**GG2**	**GG3**	**GG4**	**GG5**	**Total**
*3* + *4* = *7*	9	146	33	3	2	193
*4* + *3* = *7*	1	36	45	9	2	93
*4* + *4* = *8*	0	1	14	4	3	22
*Total*	10	183	92	16	7	308

*RP* radical prostatectomy, *GG* ISUP grade group

### Gleason pattern 4 percentage

**Fig. 2:**
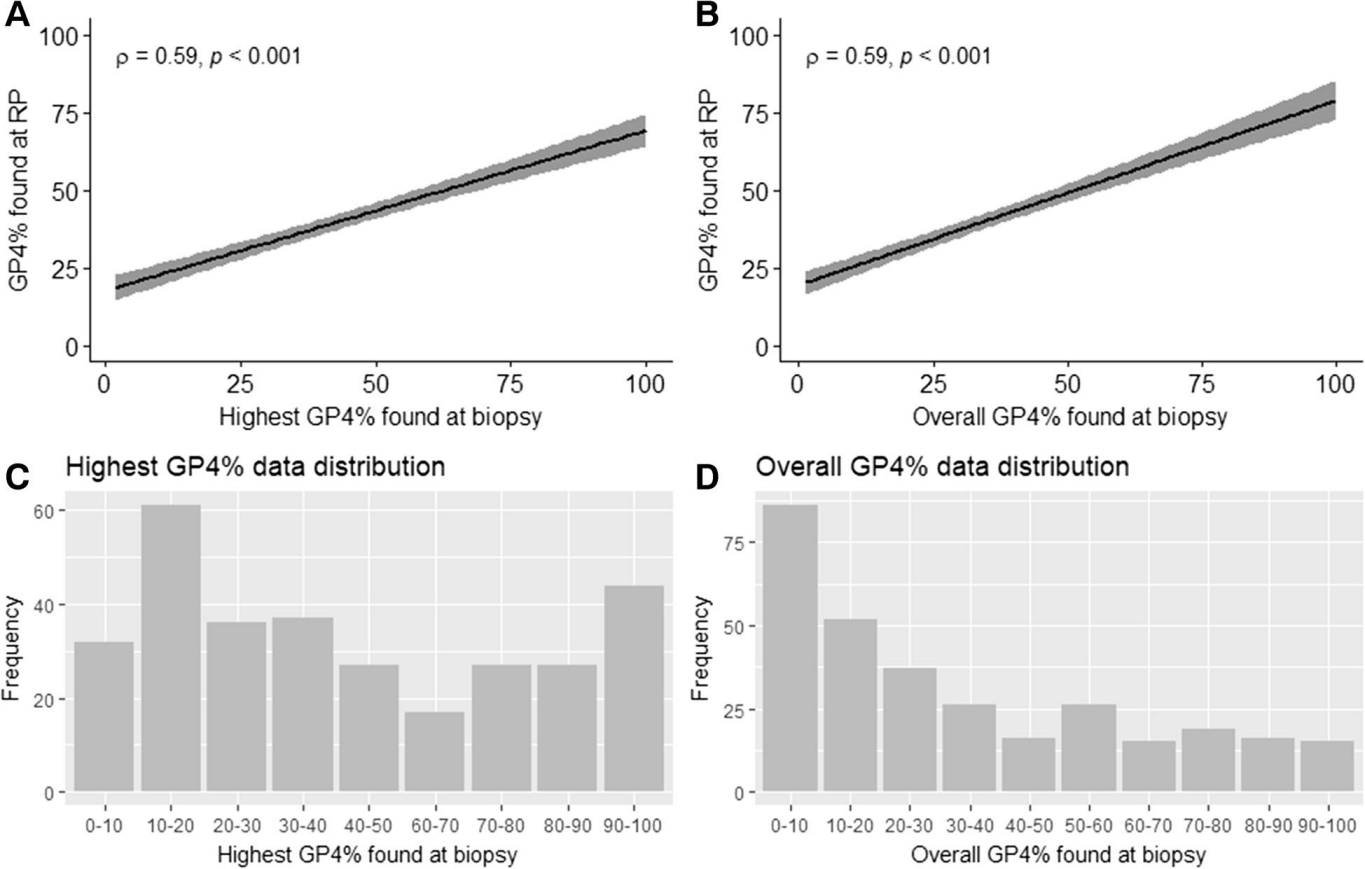
Plotted Spearman’s ranked correlation coefficient between GP4% at RP and GP4% on either the highest biopsy (**A**) or the overall biopsy (**B**), and their respective data distribution (**C**, **D**). Abbreviations: Gleason pattern 4 percentage (GP4%), rho (*ρ*), radical prostatectomy (RP)

Figure 2 illustrates the relationship between biopsy and RP GP4%. Both GP4% quantification methods were statistically significantly correlated with RP GP4% (highest rho = 0.59, overall rho = 0.59, both *p* < 0.001). No statistically significant difference between the quantification methods was found (*p* = 0.78).

### Adverse pathology at RP

After controlling for relevant clinicopathological features, both quantification methods were significantly associated with AP (per 10% increase, highest GP4% odds ratio [OR] 1.26 [95% confidence interval [CI] 1.14–1.39], overall GP4% OR 1.38 [95% CI 1.22–1.58], both *p* < 0.001), whereas CR/IDC was not (highest GP4% model, OR for CR/IDC 1.17, 95% CI 0.65–2.09, *p* = 0.6; overall model OR for CR/IDC 1.05, 95% CI 0.57–1.89, *p* = 0.9) (Table 3). The AUC for overall GP4% was slightly higher than for the highest GP4% (overall GP4% AUC 0.78, 95% CI 0.73–0.83, highest GP4% AUC 0.76, 95% CI 0.71–0.81, *p* = 0.041).

**Table 3:** Uni- and multivariable analysis of Gleason pattern 4 quantification methods and adverse pathology on radical prostatectomy. Multivariable analyses were corrected for prostate specific antigen level (PSA) (per doubling unit), clinical tumor stage (cT), percentage of tumor positive biopsies, biopsy modality, and cribriform/intraductal (CR/IDC) presence. GP4 percentages were rescaled by a factor 10 for interpretation purposes

*Analysis*	*Variable*	*OR*	*OR 95 CI%*	*p-value*	*AUC*	*AUC 95 CI%*
*Univariable highest*	Highest GP4%	1.29	1.18–1.40	< 0.001		
*Univariable overall*	Overall GP4%	1.39	1.25–1.55	< 0.001		
*Multivariable highest*	Highest GP4%	1.26	1.14–1.39	< 0.001	0.76	0.71–0.81
	PSA	1.36	1.02–1.84	0.041		
	cT					
	T1	Ref	Ref			
	T2	1.13	0.64–2.02	0.7		
	T3	2.00	0.91–4.49	0.087		
	Perc tum pos	1.03	1.01–1.04	< 0.001		
	Biopsy modality					
	Systematic	Ref	Ref			
	Target	0.97	0.33–2.98	> 0.9		
	Both	1.79	1.01–3.22	0.048		
	CR/IDC					
	Not present	Ref	Ref			
	Present	1.17	0.65–2.09	0.6		
*Multivariable overall*	Overall GP4%	1.38	1.22–1.58	< 0.001	0.78	0.73–0.83
	PSA	1.34	0.99–1.82	0.058		
	cT					
	T1	Ref	Ref			
	T2	1.07	0.99–1.92	0.8		
	T3	1.74	0.78–3.98	0.2		
	Perc tum pos	1.03	1.02–1.04	< 0.001		
	Biopsy modality					
	Systematic	Ref	Ref			
	Target	1.01	0.35–3.16	> 0.9		
	Both	2.08	1.16–3.81	0.016		
	CR/IDC					
	Not present	Ref	Ref			
	Present	1.05	0.57–1.89	0.9		

*OR* odds ratio, *CI* confidence interval, *GP4%* Gleason pattern 4 percentage, *AUC* area under the curve

## Discussion

This study compared the relation of the highest and overall GP4% at biopsy with RP GP4% and adverse pathology (pT-stage ≥ T3, GS > 4 + 3 = 7 and/or pN1). Our findings show that (a) both quantification methods are correlated with GP4% and AP at RP, and (b) overall GP4% has a statistically slightly better discriminative value for AP than the highest GP4% (AUC 0.78 versus 0.76). International genitourinary societies and the WHO recommend the reporting of GP4% in GS7 PCa biopsies, but the mode of quantification has not been specified explicitly. Although the performance of both the overall and highest GP4% is comparable, the outcome of this study slightly favors specifying overall GP4%. While the reporting of GG in biopsy specimens is optional, we believe that including an overall grade and GP4% may have added value in risk stratification and clinical decision-making in subsets of patients.

Several studies have reported on the prognostic value of biopsy GP4%, but a limited number of studies have specifically reported in-depth analyses comparing GP4% quantification methods. Dean et al. investigated whether overall GP4% and maximum GP4% in a single core could predict AP in 457 patients with biopsy GG2 [13]. AP was present in 43% of their cohort. On multivariable analysis correcting for the same clinicopathological variables as our study, except for CR/IDC, they found comparable AUC, slightly higher for maximum GP4% (0.719, 95% CI 0.652–0.785) than overall GP4% (0.697, 95% CI 0.629–0.765). Cole et al. studied the performance of overall and single core maximum GP4% and AP [17]. They included 1691 patients with GS6–8 on biopsy, < cT3 and PSA < 20 ng/ml, of whom 31% had AP, but did not specify between systematic and/or targeted biopsies. On multivariable analysis correcting for age, race, percent positive biopsy cores, cancer volume percent at biopsy, PSA, cT, and categorized GS, they found slightly higher AUC for the overall than for the highest GP4% (0.833 versus 0.826, DeLong test *p* = 0.013). Both Dean and Cole used the maximum core GP4% rather than the highest site GP4% as in our study, which could have led to higher percentages in their studies. Perera et al. studied post-operative biochemical recurrence (BCR) instead of AP [18]. They showed on the multivariable analysis of 442 GG2 patients correcting for cT, PSA, percent positive cores, and biopsy modality that overall GP4% and maximum GP4% in any single core were both associated with BCR, but were not able to identify differences between both quantification methods due to low event rate. They did report c-indices that were comparable for maximum (0.692, 95% CI 0.625–0.774) and overall GP4% (0.691, 95% CI 0.621–0.774). Sato et al. assessed 228 intermediate-risk biopsy GG2 patients and used a slightly different definition of AP (pN1, GS 4 + 3 = 7 or ≥ pT3b) [19]. They reported biopsy CR/IDC presence in only 18% of patients and AP in 24%, which were both higher in our cohort (51% and 61%, respectively). On multivariable analysis correcting for cT-stage, PSA, and percentage positive cores, they found that the highest GP4% among all cores was a predictor for AP, but did not assess nor compare to overall GP4%. They found that small and non-small cribriform patterns were not predictive for AP at multivariable analysis. A potential explanation for CR/IDC not being predictive for AP in both studies is that CR/IDC status is false negative in about half of biopsies [20]. Other studies have assessed the difference between the highest or overall biopsy GG on oncological outcomes and mainly concluded that overall GG was as good or superior compared to the highest GG [21–23].

Currently, ISUP and GUPS recommend reporting the GG per biopsy site, while the provision of an overall GG is optional. In the majority of patients, the highest and overall GG will result in similar risk stratification and treatment. However, clinically significant discrepancies may exist in a minority of cases, for instance, in men with small volume GG3 or GG4 disease at some biopsy sites and more voluminous GG1 or GG2 disease at another. In cases of grade discordances per biopsy sites, clinicians may be inclined to use the highest GG, especially when it is present in targeted biopsies. The vast majority of clinical studies do not specifically report whether the overall or highest GG was used, and guidelines generally do not distinguish between both. Based on the results of our study and others, the presence of lower grade lesions should not automatically be neglected, in particular, if the highest GP4% tumors have low volume. In that case, we recommend multidisciplinary decision-making, including consideration of MRI findings for optimal individual risk stratification.

A strength of this study was the detailed revision of all biopsies by one genitourinary pathologist. There are several limitations. Firstly, in this retrospective study, we grouped biopsies per biopsy site, rather than evaluating each individual biopsy core separately. This may have diluted higher percentages found in the highest cores, potentially leading to less difference between both quantification methods. Secondly, since the Anser Prostate Network is increasingly following men with active surveillance, our cohort might be enriched for higher-risk patients. Thirdly, the RP specimens from the centralized operation center were not available for review, which could have compensated for inter-observer effects and detailed consideration of tumor multifocality. Finally, the number of biopsies taken in this multicenter study was variable. Unlike common practice in other centers, not all patients underwent systematic biopsies with at least 12 cores. However, this heterogeneity mirrors current daily practice with different biopsy protocols among different centers.

## Conclusion

In this study, we found that the highest and overall biopsy GP4% both correlated with RP GP4%. Although the discriminative performance of the highest and overall GP4% was comparable with respect to AP at RP, the overall GP4% statistically slightly outperformed the highest GP4%. While reporting the overall GG in biopsy specimens is optional, including an overall grade and GP4% may have added value in risk stratification and clinical decision-making in a subset of patients.

## Data Availability

Data will be made available upon reasonable request.
